# Simultaneous Qualitative and Quantitative Analyses of 41 Constituents in *Uvaria macrophylla* Leaves Screen Antioxidant Quality-Markers Using Database-Affinity Ultra-High-Performance Liquid Chromatography with Quadrupole Orbitrap Tandem Mass Spectrometry

**DOI:** 10.3390/molecules29204886

**Published:** 2024-10-15

**Authors:** Xiaoqiong Xu, Xican Li, Shaoman Chen, Yongbai Liang, Chuanyang Zhang, Yuhan Huang

**Affiliations:** 1 College of Pharmacy, Gansu Medical University, Pingliang 744000, China; 2 School of Chinese Herbal Medicine, Guangzhou University of Chinese Medicine, Guangzhou 510006, China; 20221110152@stu.gzucm.edu.cn (S.C.); 20231110153@stu.gzucm.edu.cn (Y.L.); 20201110616@stu.gzucm.edu.cn (Y.H.); 3 School of Pharmacy, Chengdu University of Traditional Chinese Medicine, Chengdu 610075, China; zcyang1991@163.com

**Keywords:** *Uvaria littoralis* leaves, UHPLC-exactive-Q-Orbitrap-MS/MS, quantitation, isomer differentiation

## Abstract

To date, no study has focused on *Uvaria macrophylla* leaves with various traditional efficiencies. This paper therefore applied a database affinity ultra-high-performance liquid chromatography with quadrupole Orbitrap tandem mass spectrometry (UHPLC-Q-Orbitrap-MS/MS) strategy to analyze the lyophilized aqueous extract of *U. macrophylla* leaves. Through database comparison and MS fragment elucidation, this study has putatively identified 41 constituents belonging to flavonoid, phenolic acid, steroid, and saccharide natural product classifications. Significantly, four groups of isomers (liquiritigenin vs. isoliquiritigenin vs. pinocembrin; oroxylin A vs. wogonin vs. galangin 3-methyl ether; isoquercitrin vs. hyperoside; protocatechuic acid vs. 2,5-dihydroxybenzoic acid) have been successfully distinguished from each other. All of 41 constituents were then subjected to a quantitative analysis based on linear regression equation established by the above UHPLC-Q-Orbitrap-MS/MS strategy and an ABTS^+•^-scavenging antioxidant assay. Finally, the chemical content was multiplied by the corresponding ABTS^+•^-scavenging percentage to calculate the antioxidant contribution. It was shown that the chemical contents of 41 constituents varied from 0.003 ± 0.000 to 14.418 ± 1.041 mg/g, and gallic acid showed the highest antioxidant contribution. Gallic acid is considered as a suitable antioxidant quality-marker (Q-marker) of *U. macrophylla* leaves. These findings have scientific implications for the resource development and quality control of *U. macrophylla* leaves.

## 1. Introduction

**Figure 1:**
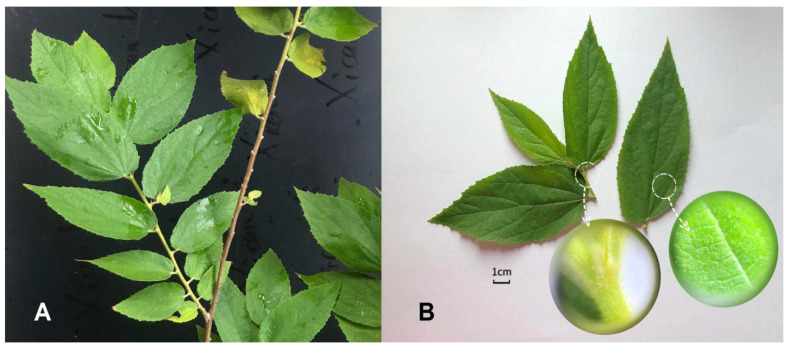
*Uvaria macrophylla* Roxb plant (aboveground, (**A**)) and its leaves (**B**). The inserted images are enlarged views (40×). All photos were taken by Xican Li in Guangzhou.

*Uvaria macrophylla* Roxburgh (Figure 1A) is a shrub in the annonaceae family and also nominated as *Uvaria littoralis*. It is mainly distributed in low-altitude hilly regions of Eastern Asia, such as the Chinese Hainan, Guangdong, and Guangxi provinces [1]. The plant mainly contains polyoxy-substituted annonaceous acetogenins, flavonoids, alkaloids, and other constituents. In traditional Chinese medicine (TCM), its leaves (Figure 1B) are used [2] for the treatment of cancer, anemia, and inflammation [3,4].

Possibly owing to their traditional effects, three plant species, *U. macrophylla*, *U. macrocarpa*, and *U. Kurzii*, have attracted interest from pharmacists and chemists. However, a substantial proportion of studies have focused on *U. microcarpa and U. Kurzii.* For example, in 2009, Yang systematically studied the chemical constituents of different parts of *U. macrocarpa* [4,5]. Liu explored the chemical constituents of *U. microcarpa* leaves [2]. Lv obtained 19 chemical constituents from another species, *U. Kurzii*, by means of conventional phytochemical approaches [3].

In contrast with *U. microcarpa*, *U. macrophylla* has received less attention from chemists. To our knowledge, only Wang’s team has focused on the constituents in the past two decades [6,7,8]. However, Wang’s work only involved the root and stem of *U. macrophylla.* Therefore, there are limitations regarding chemical studies of *U. macrophylla*, that is, no study has focused on the *U. macrophylla* leaves, which have also been documented in the resource of *Ziyupan* [9].

This study thereby attempted to analyze *U. macrophylla* leaves using a novel chemical strategy, i.e., the database affinity ultra-high-performance liquid chromatography with quadrupole Orbitrap tandem mass spectrometry (UHPLC-Q-Orbitrap-MS/MS) strategy. This strategy has been proven to be highly reliable and effective, for it could completely elucidate MS fragments and strictly discriminate different isomers [10,11,12], Meanwhile, this novel strategy was able to fulfill the quantification of these constituents [13]. Such simultaneous qualitative and quantitative analyses would bring about an in-depth understanding of *U. macrophylla* leaves, from the angle of chemistry. The in-depth chemical understanding is believed to lead to three scientific implications, i.e., clarification of the substance basis of traditional herbal medicine *Ziyupan*, the promotion of resource development of the *U. macrophylla* plant, and the establishment of quality control methods for *U. macrophylla* leaves.

## 2. Results and Discussion

**Figure 2:**
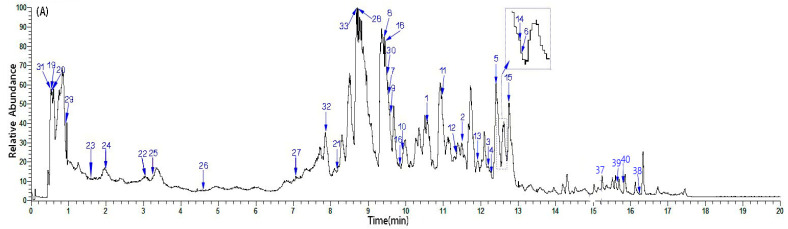

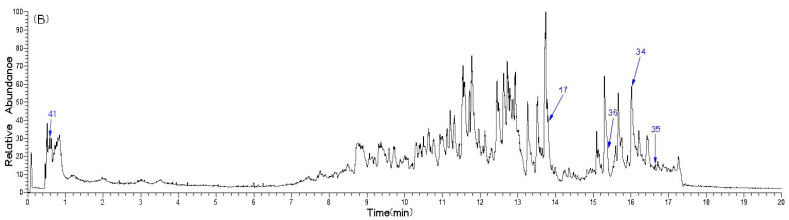
Total ion current (TIC) chromatograms of *U. macrophylla* leaves in the UHPLC-Q-Orbitrap-MS/MS analysis: (**A**) negative ion mode; (**B**) positive ion mode.

**Figure 3:**
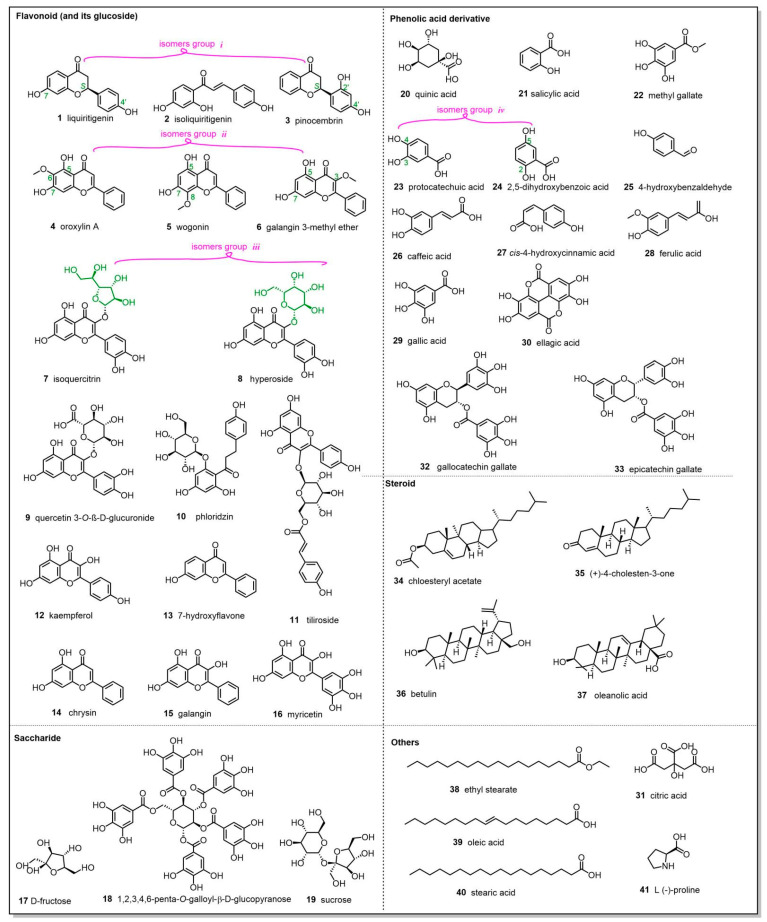
Structures and configurations of 41 identified constituents (**1**–**41**).

Sample solutions of *U. macrophylla* leaves were analyzed for constituent identification using the UHPLC-Q-Orbitrap-MS/MS strategy. Constituent identification, however, was accomplished through a comparison with authentic standards in the database and subsequent MS/MS fragment elucidation. The identification results were intuitively shown in the total ion current (TIC) diagram (Figure 2), while the structures of all identified constituents were detailed in Figure 3. The main information for constituent identification was listed in Table 1, including RT values, molecular ion peak, MS/MS fragments, observed *m*/*z* value, theoretical *m*/*z* value, and the error between the observed *m*/*z* value and theoretical *m*/*z* value.

**Figure 4:**
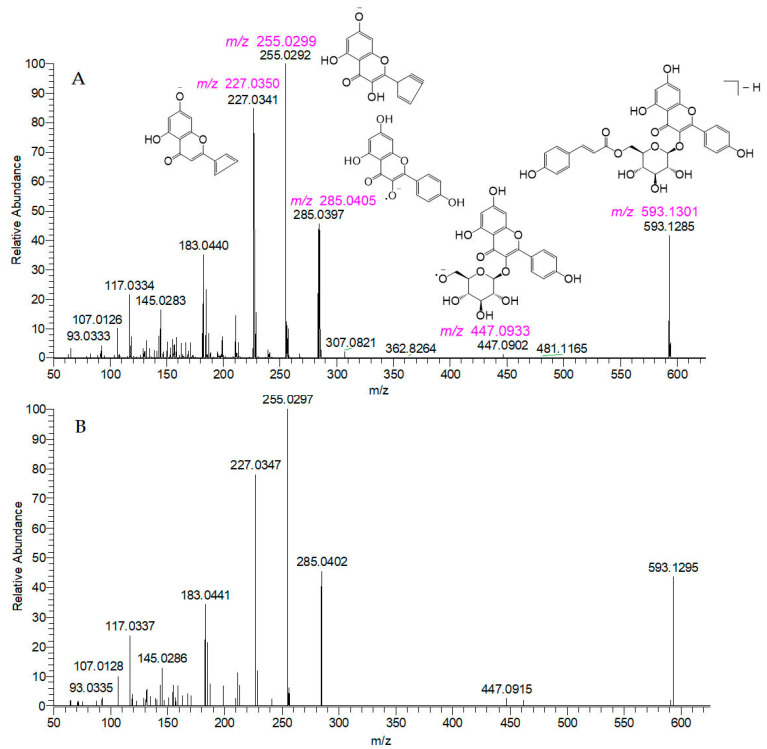
Typical MS fragments of tiliroside: (**A**) for an authentic standard of tiliroside and (**B**) for a tiliroside in *U. macrophylla* leaves sample solution (R.T. 10.96 min). The *m*/*z* values in purple are calculated based on the relative atomic mass of C (12.0000), H (1.007825), and O (15.994915).

In negative ion mode, the aqueous extract of *U. macrophylla* leaves was shown to enrich 36 constituents, most of which were flavonoids and phenolic acids. In positive ion mode, five constituents were identified as D-fructose (**17**), cholesteryl acetate (**34**), (+)-4-cholesten-3-one (**35**), betulin (**36**), and L-(-)-proline (**41**). All 41 constituents have been elucidated for their MS/MS spectra in Supplementary Materials (Suppls. S1–S41). For example, the MS elucidation spectra of tiliroside **11** are shown in Figure 4.

**Scheme 1:**
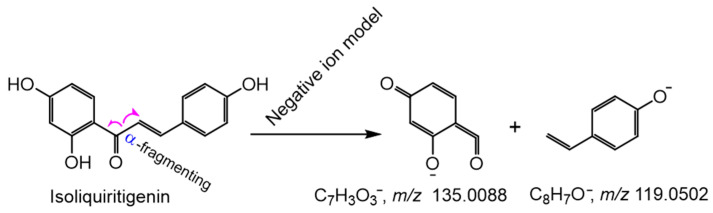
The main fragmenting pathway of isoliquiritigenin (**2**).

**Figure 5:**
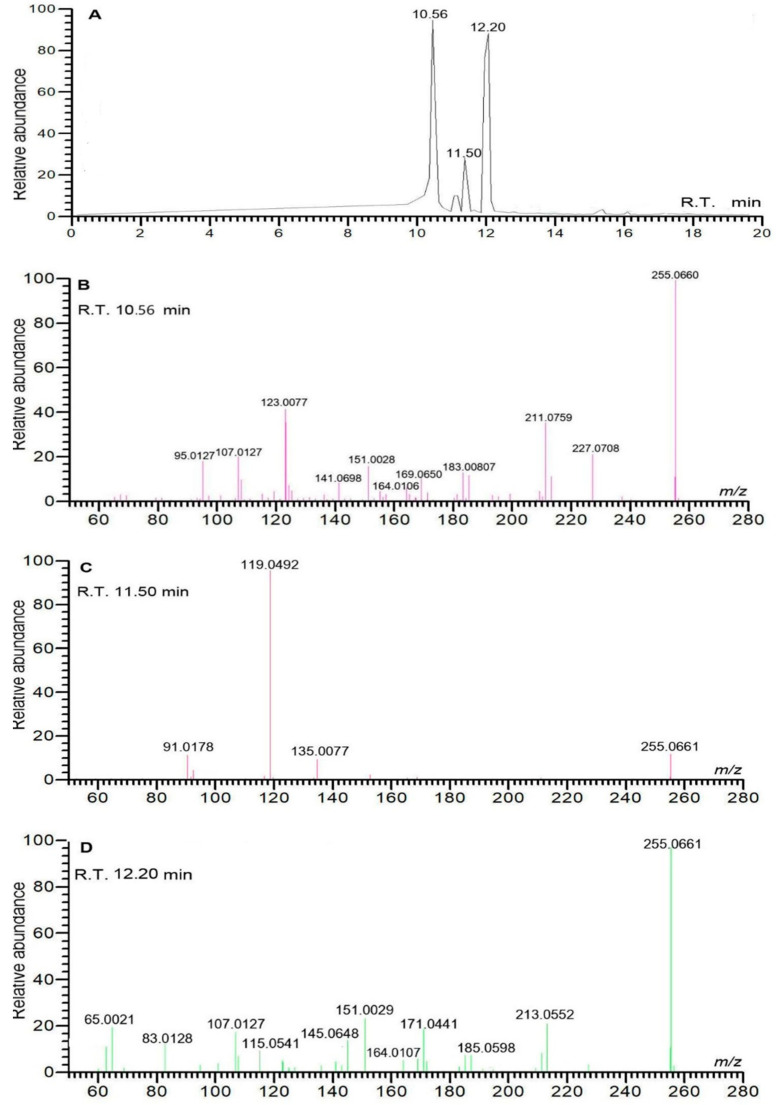
Discrimination of three isomers (liquiritigenin, isoliquiritigenin, and pinocembrin): (**A**) chromatograph for C_15_H_11_O_4_^−^ extracted using Xcalibur in negative mode; (**B**) MS/MS of peak at 10.56 min; (**C**) MS/MS of peak at 11.50 min; and (**D**) MS/MS of peak at 12.20 min.

Significantly, the constituent identification has successfully discriminated four groups of isomers. The discrimination of isomer group *i* (**1**, **2**, and **3**) is representative of the process. As seen in Figure 5, when formula C_15_H_11_O_4_^−^ was extracted using Xcalibur in negative ion mode, the chromatograms were observed to show three peaks with *m*/*z* 255. Nevertheless, their MS/MS profiles were quite different from each other because the three isomers (**1**, **2**, and **3**) formed a group of hybridized isomers rather than simple positional isomers (or functional group isomers or steric isomers). Thus, they displayed quite different fragmenting pathways, according to our previous study [14]. Isoliquiritigenin **2**, for example, underwent α-fragmentation to give two strong MS/MS peaks, *m*/*z* 135 and 119 (Scheme 1). Two other constituents, liquiritigenin **1** and pinocembrin **3,** did not give rise to these MS/MS peaks. Through comparison of these characteristic MS/MS peaks, as well as the different RT values in the database, the members in isomer group *i* were readily distinguished from each other. The identification processes and fragmentation process have been detailed in Supplementary Materials (Suppls. S1–S3).

Similarly, three members (**4**, **5**, and **6**) in isomer group *ii* constructed positional isomerism and were differentiated by their different RT values (Figure 2A and Supplementary Materials (Suppls. S4–S6)). Isomer group *iii* (**7** and **8,** Figure 3), however, included two hybridized isomers. Isomer group *iv* (**23** and **24**, Figure 3) also featured positional isomerism. Nonetheless, all these members have been completely differentiated by means of comparisons of their different RT values and MS/MS profiles. Their MS/MS fragments have been elucidated in Supplementary Materials (Suppls. S4–S8 and S23–S24). The success in the differentiation of these isomers has implied that the database-aided UHPLC-Q-Orbitrap-MS/MS was a reliable strategy.

Using the reliable database-aided UHPLC-Q-Orbitrap-MS/MS strategy, this study has putatively identified 41 constituents in *U. macrophylla* leaves. Subsequently, the contents of all 41 identified constituents (**1**–**41**) were also quantitatively analyzed using the database-aided UHPLC-Q-Orbitrap-MS/MS strategy. As seen in Table 1, their chemical contents varied from 0.003 ± 0.000 to 14.418 ± 1.041 mg/g. One steroid, cholesteryl acetate (**34**), displayed the highest chemical content, while another steroid, (+)-4-cholesten-3-one (**35**), exhibited the lowest chemical content.

From the angle of pharmacology, these constituents showed various beneficial effects, such as antitumor effects (e.g., pinocembrin), anti-inflammatory effects (e.g., wogonin), neuroprotection (e.g., gallocatechin gallate), and cardiovascular protection (e.g., citric acid). In particular, five constituents are actually nutrients that can improve anemia [15], including sucrose, 2,5-dihydroxybenzoic acid, 4-hydroxybenzaldehyde, and L-(-)-proline. Nine constituents have been reported to be natural antioxidants [15], such as gallic acid and ellagic acid (Table 1). These beneficial effects are relevant to the aforementioned traditional efficiencies of *Ziyupan*. Nevertheless, there is not a certain bioactivity to effectively characterize the traditional efficiencies of *Ziyupan*. Thereby, this study had to comparatively evaluate their relative antioxidant abilities, because antioxidation plays an indispensable role in these traditional efficiencies and bioactivities [16,17,18].

**Figure 6:**
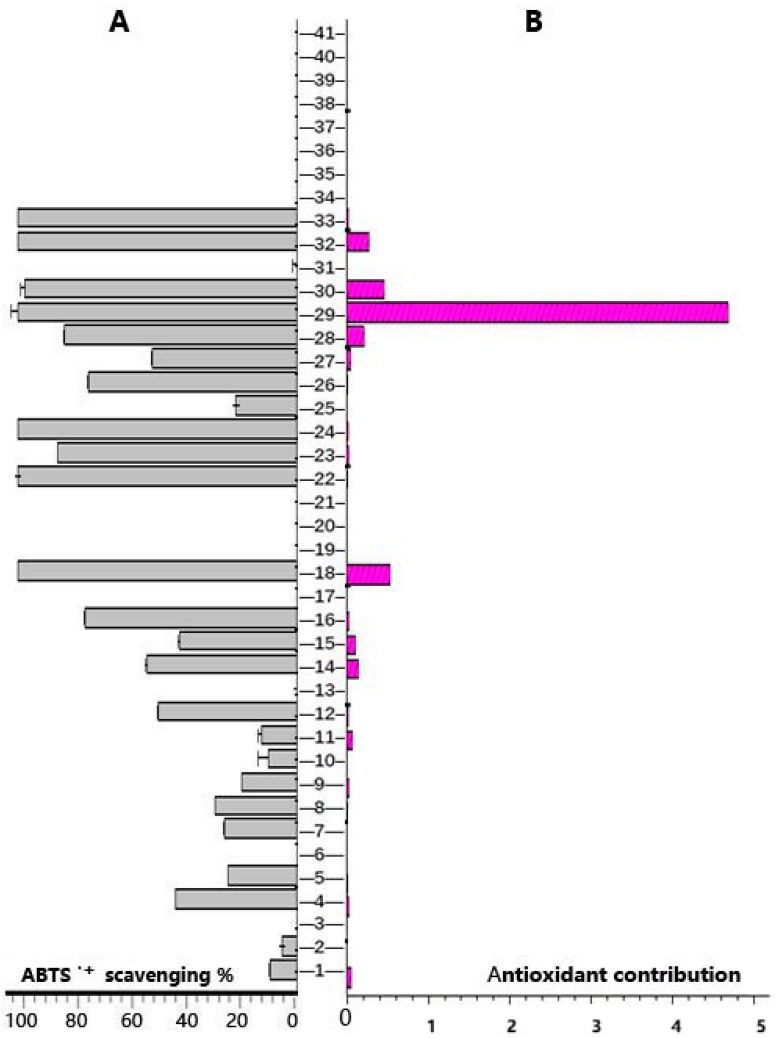
Relative antioxidant levels of 41 components (**1**–**41,** (**A**)) and corresponding antioxidant contributions (**B**). The relative antioxidant level was evaluated using an ABTS^•+^-scavenging assay.

To guarantee comparability, all 41 constituents were individually analyzed using an ABTS^+•^-scavenging antioxidant assay at the same dose (3 μL × 0.5 μg/μL). As seen in Figure 6A, these constituents showed different antioxidant levels in the ABTS^+•^-scavenging assay. Some constituents (e.g., **34**–**41**) exhibited negligible ABTS^+•^-scavenging percentages, while six constituents (e.g., **18**, **22**, **24**, **29**, **32**, and **33**) exhibited 100% ABTS^+•^-scavenging percentages. However, the six showed different chemical contents, as indicated in Table 1. Therefore, this study defined a new formula (Equation (1)) to calculate the antioxidant contribution and then characterize the role of one antioxidant constituent in whole *U. macrophylla* leaves.

Antioxidant contribution = relative antioxidant level × chemical content(1)

In line with the calculation, gallic acid (**29**) possessed the highest antioxidant contribution value, which was also much higher than the sum of antioxidant contribution values of other constituents (Figure 6B). Therefore, gallic acid (**29**) was considered to be the most antioxidant contributor in *U. macrophylla* leaves.

Herein, this study used a novel database-aided UHPLC-Q-Orbitrap-MS/MS strategy to simultaneously qualitatively and quantitatively analyze 41 constituents in *U. macrophylla* leaves. These constituents covered several natural product classifications, including flavonoids, phenolic acids, steroids, and saccharides. Particularly, the qualitative analysis has successfully discriminated 10 isomeric constituents to accomplish a putative identification of all 41 constituents. Thereafter, all 41 constituents were quantitatively analyzed using the abovementioned database-aided UHPLC-Q-Orbitrap-MS/MS strategy. Finally, these constituents were evaluated for the relative antioxidant level using an ABTS^•+^-scavenging assay to calculate their individual antioxidant contributions. One phenolic acid, gallic acid, showed substantial chemical content and extremely high antioxidant contribution toward *U. macrophylla* leaves. Therefore, it was recommended as the antioxidant quality marker (Q-marker) of *U. macrophylla* leaves. Apparently, all these in-depth chemical understandings have not only facilitated the resource development and quality control of *U. macrophylla* leaves but also helped to clarify the substance basis of the traditional herbal medicine *Ziyupan*.

**Table 1:** Main experimental data and documental bioactivity of 41 constituents (**1**–**41**) in *U. macrophylla* leaves.

No.	RT Min	Name	Molecular Ion	Observed *m*/*z* Value	Theoretical *m*/*z* Value	Error (δ ppm)	Content (mg/g) n = 3	Characteristic MS/MS Fragment Peak *m*/*z*	Bioactivity
**1**	10.56	liquiritigenin	C_15_H_11_O_4_^−^	255.0661	255.0663	0.7843	0.577 ± 0.013	255.0659, 119.0492, 91.0178	anti-inflammatory [19]
**2**	11.50	isoliquiritigenin	C_15_H_11_O_4_^−^	255.0661	255.0663	0.7843	0.012 ± 0.000	255.0661, 119.0492, 91.0178	antitumor [20]
**3**	12.20	pinocembrin	C_15_H_11_O_4_^−^	255.0662	255.0663	0.3921	0.072 ± 0.000	255.0661, 213.0552, 151.0029	antitumor [21]
**4**	12.29	oroxylin A	C_16_H_11_O_5_^−^	283.0612	283.0612	0	0.048 ± 0.002	283.0611, 268.0377	antioxidant [22]
**5**	12.35	wogonin	C_16_H_11_O_5_^−^	283.0611	283.0612	0.3533	0.026 ± 0.001	283.0611, 163.0027, 268.0374 109.9998	anti-inflammatory [23]
**6**	12.52	galangin 3-methyl ether	C_16_H_11_O_5_^−^	283.0612	283.0612	0	0.012 ± 0.000	239.0346, 211.0395, 167.0494	antibiotic [24]
**7**	9.54	isoquercitrin	C_21_H_19_O_12_^−^	463.0873	463.0882	1.9438	0.017 ± 0.002	463.0874, 300.0273, 271.0247 255.0298	anti-inflammatory [25]
**8**	9.43	hyperoside	C_21_H_19_O_12_^−^	463.0875	463.0882	1.5118	0.025 ± 0.002	463.0874, 300.0273, 271.0247	anti-inflammatory [26]
**9**	9.57	quercetin 3-O-β-D-glucuronide	C_21_H_17_O_13_^−^	477.0674	477.0675	0.2096	0.138 ± 0.005	301.0353, 151.0028, 109.0284	antimicrobial [27]
**10**	9.93	phloridzin	C_21_H_23_O_10_^−^	435.1280	435.1297	3.9080	0.034 ± 0.001	273.0770, 167.0341, 123.0442, 119.0492	antioxidant [28,29]
**11**	10.96	tiliroside	C_30_H_25_O_13_^−^	593.1298	593.1301	0.5059	0.542 ± 0.007	285.0397, 255.0292, 227.0341	antioxidant [30]
**12**	11.36	kaempferol	C_15_H_9_O_6_^−^	285.0405	285.0405	0	0.033 ± 0.000	285.0405, 117.0335, 93.0334	anti-inflammatory [31]
**13**	11.93	7-hydroxyflavone	C_15_H_9_O_3_^−^	237.0553	237.0557	1.6877	0.027 ± 0.000	237.0553, 208.0524, 91.0178	antibacterial [32]
**14**	12.51	chrysin	C_15_H_9_O_4_^−^	253.0504	253.0506	0.7905	0.267 ± 0.002	251.0500, 209.0598, 143.0491	antitumor [33]
**15**	12.67	galangin	C_15_H_9_O_5_^−^	269.0456	269.0455	0.3717	0.247 ± 0.001	269.0456, 169.0650	anti-inflammatory [34]
**16**	9.86	myricetin	C_15_H_9_O_8_^−^	317.0301	317.0303	0.6309	0.036 ± 0.005	317.0301, 151.0028, 137.0234, 109.0284	antioxidant [35]
**17**	13.82	D-fructose	C_6_H_13_O_6_^+^	181.0715	181.0707	4.4198	0.247 ± 0.000	163.0385, 149.0229, 65.0393	antioxidant [36]
**18**	9.46	1,2,3,4,6-penta-*O*-galloyl-β-D-glucopyranose	C_41_H_31_O_26_^−^	939.1146	939.1109	3.9403	0.547 ± 0.700	169.0134, 125.0234, 107.0127, 95.0126	antioxidant [37]
**19**	0.53	sucrose	C_12_H_21_O_11_^−^	341.1086	341.1089	0.8797	0.986 ± 0.048	341.1086, 179.0553, 161.0448, 89.0233	sweetening agent [38]
**20**	0.55	quinic acid	C_7_H_11_O_6_^−^	191.0555	191.0561	3.1413	0.377 ± 0.003	191.0555, 93.0335, 85.0283	antioxidant [39]
**21**	8.18	salicylic acid	C_7_H_5_O_3_^−^	137.0234	137.0244	7.2992	0.056 ± 0.001	137.0234, 93.0333	anti-inflammatory [40,41]
**22**	3.06	methyl gallate	C_8_H_7_O_5_^−^	183.0291	183.0299	4.3715	0.007 ± 0.000	183.0291, 124.0156, 78.0099	anti-inflammatory [42]
**23**	1.6	protocatechuic acid	C_7_H_5_O_4_^−^	153.0186	153.0193	4.5751	0.026 ± 0.006	153.0186, 109.0285, 108.0206	antimicrobial [43]
**24**	2	2,5-dihydroxybenzoic acid	C_7_H_5_O_4_^−^	153.0186	153.0193	4.5751	0.015 ± 0.003	153.0182, 109.0284, 108.0206	improvements in vascular function [44]
**25**	3.22	4-hydroxybenzaldehyde	C_7_H_5_O_2_^−^	121.0285	121.0295	8.2644	0.006 ± 0.001	121.0285, 92.0257	improvements in vascular function [45,46]
**26**	4.6	caffeic acid	C_9_H_7_O_4_^−^	179.0339	179.0350	6.1452	0.012 ± 0.000	179.0345, 136.0474,135.0441, 133.0282	antibacterial [47]
**27**	7.11	*cis*-4-Hydroxycinnamic acid	C_9_H_7_O_3_^−^	163.0392	163.0401	5.5214	0.094 ± 0.005	119.0492	anti-SARS [48]
**28**	8.71	ferulic acid	C_10_H_9_O_4_^−^	193.0496	193.0506	5.1813	0.258 ± 0.301	193.0136, 178.0262, 134.0364	antibacterial [49,50]
**29**	0.86	gallic acid	C_7_H_5_O_5_^−^	169.0133	169.0142	5.3254	4.800 ± 0.103	169.0133, 125.0234	antioxidant [51]
**30**	9.5	ellagic acid	C_14_H_5_O_8_^−^	300.9989	300.9990	0.3333	0.485 ± 0.000	300.9990, 145.0286, 117.0335	antioxidant [52]
**31**	0.52	citric acid	C_6_H_7_O_7_^−^	191.0189	191.0197	4.1884	0.757 ± 0.007	111.0078, 87.0076, 85.0248	improvements in vascular function [53]
**32**	7.98	gallocatechin gallate	C_22_H_17_O_11_^−^	457.0736	457.0776	8.7527	0.274 ± 0.004	169.0133, 125.0234	antiviral [54]
**33**	8.68	epicatechin gallate	C_22_H_17_O_10_^−^	441.0831	441.0827	0.9070	0.020 ± 0.000	289.0715, 169.0134, 125.0234, 109.0285	antioxidant [55]
**34**	16.03	cholesteryl acetate	C_29_H_49_O_2_^+^	429.3706	429.3727	4.8951	14.418 ± 1.041	429.3706, 165.0912, 91.0545, 81.0705,	antitumor [56]
**35**	16.67	(+)-4-cholesten-3-one	C_27_H_45_O^+^	385.3459	385.3465	1.5584	0.003 ± 0.000	385.3454, 109.0649, 97.0650, 91.0545	antitumor [57]
**36**	15.38	betulin	C_30_H_51_O_2_^+^	443.3878	443.3884	1.3544	0.130 ± 0.005	443.3497, 105.0700, 91.0547, 81.0705	antitumor [58]
**37**	15.18	oleanolic acid	C_30_H_47_O_3_^−^	455.3531	455.3531	0	0.026 ± 0.004	455.3531	antitumor [59]
**38**	16.22	ethyl stearate	C_20_H_39_O_2_^−^	311.2956	311.2956	0	0.209 ± 0.013	311.1682, 183.0114, 119.0492	antioxidant [60]
**39**	15.62	oleic acid	C_18_H_33_O_2_^−^	281.2487	281.2486	0.3558	0.046 ± 0.000	281.2487	antioxidant [61]
**40**	15.8	stearic acid	C_18_H_35_O_2_^−^	283.2643	283.2643	0	0.197 ± 0.002	283.2643, 92.1626	antioxidant [62]
**41**	0.54	L-(-)-proline	C_5_H_10_NO_2_^+^	116.0708	116.0706	1.7241	0.940 ± 0.045	116.0706, 70.0657	immune modulation [63]

Note: *m*/*z* values below 50 were also found using the Xcalibur 4.1 software package, despite the scan mode range of *m*/*z* 100–1500. *m*/*z* values in square brackets are the molecular ion peaks. Nominal level (NL) values were obtained using the Xcalibur 4.1 software package. Five compounds (**17**, **34**, **35**, **36**, and **41**) annotated with “^+^” under *Molecular ion* were identified in positive ion mode. All other compounds were identified in negative ion mode.

## 3. Materials and Methods

### 3.1. Plant Materials and Chemicals

The *U. macrophylla* leaves (10 g) were clipped at Yaowang Mountain, Guangzhou University of Chinese Medicine (23.039° N, 113.388° E, altitude 20 m, Guangzhou, China) on 12 July 2023. Samples were identified as genuine by Professor Wang Xifang of the Shaanxi University of Chinese Medicine, dried at low temperature, placed into a self-sealing bag and labeled as “*Uvaria macrophylla* Roxburgh”, and refrigerated (2–8 °C). The methanol and water used for the preparation and analysis of the sample solution were of MS purity (Merck Sigma-Aldrich, Shanghai, China).

### 3.2. Authentic Standards

Liquiritigenin (CAS 578-86-9, C_15_H_12_O_4_, MW 256.25, 98%), isoliquiritigenin (CAS 961-29-5, C_15_H_12_O_4_, MW 256.25, 98%), pinocembrin (CAS 480-39-7, C_15_H_12_O_4_, MW 256.25, 98%), oroxylin A (CAS 480-11-5, C_16_H_12_O_5_, MW 284.26, 98%), wogonin (CAS 632-85-9, C_16_H_12_O_5_, MW 284.26, 98%), galangin 3-methyl ether (CAS 6665-74-3, C_16_H_12_O_5_, MW 284.26, 98%), isoquercitrin (CAS 21637-25-2, C_21_H_20_O_12_, MW 464.38, 98%), and hyperoside (CAS 482-36-0, C_21_H_20_O_12_, MW 464.38, 98%) were obtained from Shanghai Maclin Biochemical Technology Co., Ltd. (Shanghai, China). Quercetin 3-*O*-β-D-glucuronide (CAS 22688-79-5, C_21_H_18_O_13_, MW 478.36, 98%), phloridzin (CAS 60-81-1, C_21_H_24_O_10_, MW 436.13, 98%), tiliroside (CAS 20316-62-5, C_30_H_26_O_13_, MW 594.52, 98%), kaempferol (CAS 520-18-3, C_15_H_10_O_6_, MW 286.24, 98%), 7-hydroxyflavone (CAS 6665-86-7, C_15_H_10_O_3_, MW 238.24, 98%), chrysin (CAS 480-40-0, C_15_H_10_O_4_, MW 254.24, 99%), galangin (CAS 548-83-4, C_15_H_10_O_5_, MW 270.24, 98%), myricetin (CAS 529-44-2, C_15_H_10_O_8_, MW 318.24, 98%), D-fructose (CAS 57-48-7, C_6_H_12_O_6_, MW 180.16, 98%), and 1,2,3,4,6-penta-*O*-galloyl-β-D-glucopyranose (CAS 14937-32-7, C_41_H_32_O_26_, MW 940.68, 98%) were obtained from Merck-Sigma Co., Ltd. (Shanghai, China). Sucrose (CAS 57-50-1, C_12_H_22_O_11_, MW 342.3, 99.5%), quinic acid (CAS 77-95-2, C_7_H_12_O_6_, MW 192.17, 98%), salicylic acid (CAS 69-72-7, C_7_H_6_O_3_, MW 138.12, 99.5%), methyl gallate (CAS 99-24-1, C_8_H_8_O_5_, MW 184.15, 99%), protocatechuic acid (CAS 99-50-3, C_7_H_6_O_4_, MW 154.12, 99%), 2,5-dihydroxybenzoic acid (CAS 490-79-9, C_7_H_6_O_4_, MW 154.12, 99%), 4-hydroxybenzaldehyde (CAS 123-08-0, C_7_H_6_O_2_, MW 122.12, 98%), and caffeic acid (CAS 331-39-5, C_9_H_8_O_4_, MW 180.16, 99%) were purchased from Sichuan Weikeqi Biological Technology Co., Ltd. (Chengdu, China). Cis-4-hydroxycinnamic acid (CAS 4501-31-9, C_9_H_8_O_3_, MW 164.16, 99%), ferulic acid (CAS 1135-24-6, C_10_H_10_O4, MW 194.18, 98%), gallic acid (CAS 149-91-7, C_7_H_6_O_5_, MW 170.12, 98%), ellagic acid (CAS 476-66-4, C_14_H_6_O_8_, MW 302.19, 98%), citric acid (CAS 77-92-9, C_6_H_8_O_7_, MW 192.12, 99.5%), gallocatechin gallate (CAS 4233-96-9, C_22_H_18_O_11_, MW 458.37, 99%), epicatechin gallate (CAS 1257-08-5, C_22_H_18_O_10_, MW 442.37, 99%), cholesteryl acetate (CAS 604-35-3, C_29_H_48_O_2_, MW 428.69, 98%), (+)-4-cholesten-3-one (CAS 601-57-0, C_27_H_44_O, MW 384.64, 98%), betulin (CAS 473-98-3, C_30_H_50_O_2_, MW 442.72, 98%), oleanolic acid (CAS 508-02-1, C_30_H_48_O_3_, MW 456.7, 98%), ethyl stearate (CAS 111-61-5, C_20_H_40_O_2_, MW 312.53, 98%), oleic acid (CAS 112-80-1, C_18_H_34_O_2_, MW 282.46, 98%), stearic acid (CAS 57-11-4, C_18_H_36_O_2_, MW 284.48, 98%), and L-(-)-proline (CAS 147-85-3, C_5_H_9_NO_2_, MW 115.13, 98%) were obtained from Shanghai Yuanye Biotechnology Co., Ltd. (Shanghai, China).

### 3.3. Preparation of Sample and Authentic Standard Solutions

#### 3.3.1. Preparation of Lyophilized Aqueous Extract and Sample Solutions

**Figure 7:**
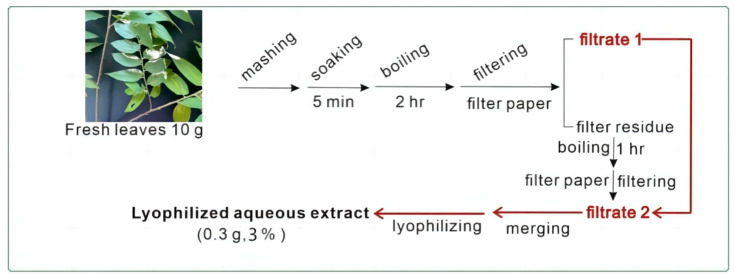
Flowchart for preparation of lyophilized aqueous extract of *U. macrophylla* leaves.

The extraction process is depicted in Figure 7. To avoid possible insoluble impurities and solvent effects [64], 10.0 g of *U. macrophylla* leaf powder was accurately weighed and placed in a round-bottomed flask with a stopper. After soaking for 5 min in 50 mL water, boiling extraction was carried out twice, the first time for 2 h and the second for 1 h. After cooling and filtering, the filtrates were lyophilized according to a previous report [65] to yield 0.3 g brown lyophilized powder. The powder was re-dissolved using 80% methanol at 30 mg/mL concentration. The methanolic solution was filtered using a 0.45 μm organic filter membrane to afford the sample solution, which was stored in the refrigerator at 2–8 °C for further analysis.

#### 3.3.2. Preparation of Authentic Standard Solutions

All authentic standards listed in Section 3.2 were dissolved in methanol at 30 μg/mL concentration and filtered through a 0.45 μm membrane. Filtrates were stored at 2–8 °C for further analysis [66].

### 3.4. Simultaneous Qualitative and Quantitative Analyses Using Database Affinity UHPLC-Q-Orbitrap-MS/MS

The main operations of injection, data acquisition, and analysis were controlled using the Xcalibur 4.1 package (Thermo Fisher Scientific Inc., Waltham, MA, USA) comprising TraceFinder 4.1, mzVault, and FreeStyle, viewing software, with the UHPLC-Q-exactive-Orbitrap-MS apparatus. Before data acquisition, the background sign was zeroed using a blank solvent. The acquired data were then exported to TraceFinder for *m*/*z* extraction and to afford the corresponding MS spectra [67]. The main parameters were set as follows: mass range 100–1500 Da; mass tolerance 10 ppm; S/N threshold 5; and isotopic pattern fit threshold 90%. The MS spectra and corresponding top 5 secondary spectra were screened using Xcalibur 4.1. By comparing 4 parameters with authentic standards (retention time, molecular ion peak, MS/MS profile, and characteristic fragments), the constituents were preliminarily nominated by TraceFinder and were putatively identified manually [15].

Quantitative analysis of the 41 identified constituents was carried out according to a published method with minor modifications [68]_._ In brief, linear regression equations were first established using authentic standard solutions. TraceFinder and Xcalibur 4.1 software offered peak area parameters for these authentic standard solutions. *U. macrophylla* leaves sample solutions were subsequently analyzed under the same chromatography and MS spectra conditions. In line with the linear regression equations and the peak areas of the identified constituents, the contents of the *U. macrophylla* leaves constituents were quantified and expressed as mean ± SD (standard deviation).

### 3.5. Relative Antioxidant Level Evaluation Experiment

The relative antioxidant level was evaluated using an ABTS^+•^-scavenging assay [69], with some modifications. ABTS^+•^ was produced by mixing 700 μL of (NH_4_)_2_ABTS aqueous solution (7.4 mmol/L) with 700 μL of K_2_S_2_O_8_ aqueous solution (2.6 mmol/L). The mixture was incubated in the dark at room temperature for 12 h. Thereafter, the incubated mixture was diluted with methanol (at a ratio of approximately 1:15) so that its absorbance at 734 nm was measured to be 0.65 ± 0.01 using a microplate reader (Multiskan FC, Thermo Scientific, Shanghai, China). Then, 3 μL of sample solution (0.5 μg/μL) was added to 17 μL of methanol and treated with 80 μL of ABTS^+•^ reagent to determine the scavenging activity. The absorbance at 734 nm was measured using the above microplate reader after initial mixing. After a 6 min incubation, the absorbance at 734 nm was measured. The ABTS^+•^-scavenging percentage was calculated using Equation (2).

(2) $$Scavenging\%=\frac{A_{0}-A}{A_{0}}\times 100\%$$

where *A*_0_ is the absorbance at 734 nm of the control (reaction system without sample), and A is the absorbance at 734 nm of the reaction mixture with the sample. Methanol was used as the blank group.

### 3.6. Statistical Analysis

Each quantitative analysis experiment and each ABTS^+•^-scavenging assay experiment were performed in triplicate. The data are shown as the mean ± SD from three independent measurements. Correlation coefficients (R values) were calculated using linear analysis with Origin 6.0 professional software (Origin-Lab Corporation, Northampton, MA, USA).

## 4. Conclusions

*U. macrophylla* leaves contain at least 41 constituents (including 10 isomers). These constituents belong to several natural product classifications, such as flavonoids, phenolic acids, steroids, and saccharides. They show different chemical contents and antioxidant contributions. Gallic acid has the highest antioxidant contribution and is applicable to act as the antioxidant Q-marker of *U. macrophylla* leaves.

## Data Availability

All the data used to support the findings of this study are available from the corresponding author upon request.

## Supplementary Materials

The following supporting information can be downloaded at https://www.mdpi.com/article/10.3390/molecules29204886/s1: Suppls. S1–S41: UHPLC-Q-Orbitrap-MS spectra and identification of **1**–**41**. Reference [70] are cited in the supplementary materials file
