# Supplementary material for: Simultaneous Qualitative and Quantitative Analyses of 41 Constituents in Uvaria macrophylla Leaves Screen Antioxidant Quality-Markers Using Database-Affinity Ultra-High-Performance Liquid Chromatography with Quadrupole Orbitrap Tandem Mass Spectrometry
Source: Molecules. 2024 Oct 15;29(20):4886. doi: 10.3390/molecules29204886 (PMC11510267; doi:10.3390/molecules29204886)
Supplement: Supplementary file 1 [file molecules-29-04886-s001.zip › Suppl. S33 epicatechin gallate CAS 1257-08-5.pdf]

Suppl. 33 Identification of epicatechin gallate (CAS 1257-08-5, $C_{22}H_{18}O_{10}$, M.W. 442.37)

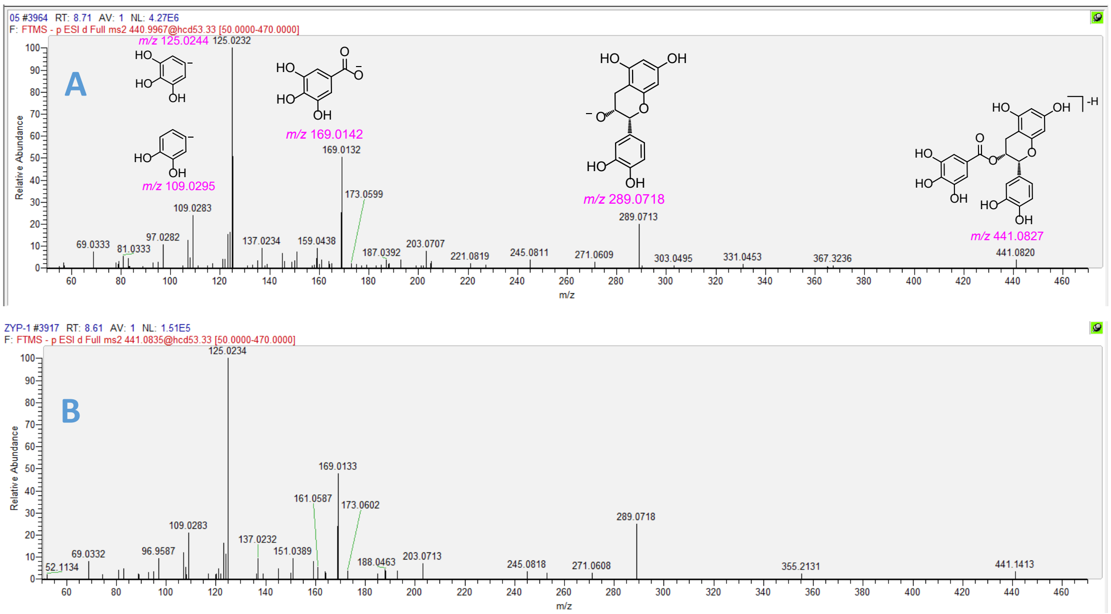

Fig. S33 The main results of standard epicatechin gallate (CAS 1257-08-5, $C_{22}H_{18}O_{10}$) and its corresponding peak in the TIC diagram using UPLC-Q-Orbitrap-MS analysis. (**A**) The MS/MS fragments of standard epicatechin gallate. (**B**) The MS/MS spectra from chromatographic peak in the *Uvaria macrophylla Roxburg* leaves extract.

Note: The *m/z* values in purple are the calculated ones. The *m/z* calculation was based on the relative atomic masses of C (12.0000), H (1.007825), O (15.994915), and N (14.003074)[1].

Identification: As seen in Fig. S33, the R.T. value, molecular ion peak, MS/MS spectra, and characteristic pears were highly similar. Thus, the chromatographic peak in the in the *Uvaria macrophylla Roxburg* leaves extract was identified as epicatechin gallate (CAS 1257-08-5, $C_{22}H_{18}O_{10}$).

References

[1] Gross., J.H. Mass spectrometry, *Beijing: Science press*. **2013**.
